# Chemical Composition and Antibacterial and Cytotoxic Activities of *Allium hirtifolium* Boiss

**DOI:** 10.1155/2013/696835

**Published:** 2012-12-23

**Authors:** Salmiah Ismail, Farid Azizi Jalilian, Amir Hossein Talebpour, Mohsen Zargar, Kamyar Shameli, Zamberi Sekawi, Fatemeh Jahanshiri

**Affiliations:** ^1^Department of Microbiology, Faculty of Biotechnology and Biomolecular Sciences, Universiti Putra Malaysia, Serdang, 43400 Selangor, Malaysia; ^2^Department of Microbiology, Faculty of Medicine, Ilam University of Medical Sciences (IUMS), Ilam, Iran; ^3^Jahad-Keshavarzi Research Center, Tabriz, Iran; ^4^Department of Biology, Faculty of Basic Sciences, Azad Islamic University, Qom Branch, Qom, Iran; ^5^Department of Chemistry, Faculty of Sciences, Universiti Putra Malaysia, Serdang, Selangor, Malaysia; ^6^Department of Medical Microbiology and Parasitology, Faculty of Medicine and Health Sciences, Universiti Putra Malaysia, Serdang, Selangor, Malaysia

## Abstract

*Allium hirtifolium* Boiss. known as Persian shallot, is a spice used as a traditional medicine in Iran and, Mediterranean region. In this study, the chemical composition of the hydromethanolic extract of this plant was analyzed using GC/MS. The result showed that 9-hexadecenoic acid, 11,14-eicosadienoic acid, and n-hexadecanoic acid are the main constituents. The antibacterial activity of the shallot extract was also examined by disk diffusion and microdilution broth assays. It was demonstrated that Persian shallot hydromethanolic extract was effective against 10 different species of pathogenic bacteria including methicillin resistant *Staphylococcus aureus* (MRSA), methicillin sensitive *Staphylococcus aureus* (MSSA), *Staphylococcus aureus*, *Staphylococcus epidermidis*, *Streptococcus pneumoniae*, *Escherichia coli, Escherichia coli* O157:H7, *Salmonella typhimurium, Proteus mirabilis*, and *Klebsiella pneumoniae*. Specifically, the minimum concentration of the extract which inhibited bacterial growth (MIC values) was 1.88 mg/mL for most of the gram-positive bacteria. This concentration was not much different from the concentration that was safe for mammalian cells (1.50 mg/mL) suggesting that the hydromethanolic extract of Persian shallot may be a safe and strong antibacterial agent.

## 1. Introduction

The problems of multidrug resistances exhibited by human pathogenic microorganisms and the side effects of antibiotics have led scientists to search for alternatives such as medicinal plants. The phenolic compounds present in most plants have a broad spectrum of biological activities whereby their antimicrobial actions stand out [1, 2].

The use of *Allium* genus members such as garlic and onion in the treatment of various ailments has been reported worldwide. Many members in this genus have been proved to possess antibacterial, antifungal, antiprotozoal, and anthelmintic activities [3–6]. In addition, *Allium* plants are believed to heal diabetes, arthritis, colds and flu, stress, fever, coughs, headache, hemorrhoids, asthma, arteriosclerosis, cancer, rheumatic, and inflammatory disorders [7–12].

*Allium hirtifolium *Boiss. (Persian shallot) is one of the Iranian native spices [6, 13] which belong to the same biological genus of *Allium sativum* (garlic) and other onions [14]. It is one of the valuable members of *Allium* with its bulbs commonly used as a traditional remedy [14, 15]. Besides, the dried bulb slices are used as additives to yogurt as well as pickling mixtures. The Persian shallot (also known as “mooseer”) is different from the common shallot (*Allium ascalonicum*) in many characteristics. For instance, the bulbs of common shallot are pear shaped with the skin reddish brown in color and its cluster may contain as many as 15 bulbs [16], while for the Persian shallot its bulbs are oval shaped and have white color of skin and normally consists of single or sometimes two bulbs [17]. In this work, we report the chemical composition and antibacterial as well as cytotoxic effects of this spice. 

## 2. Materials and Methods

### 2.1. Plant Material

The bulbs of *Allium hirtifolium* Boiss were collected from Ilam Province of Iran during September to October 2009. The plant specimen (number CMN10) was deposited in the Herbarium of Materia Medica, Research Center of Agriculture and Natural Sources (RCANS) of Ilam Province.

### 2.2. Extract Preparation

The bulbs of Persian shallot were washed with tap water and cut into small slices. The air-dried bulbs were grinded into powder by using a blender. The extraction was carried out using percolation procedure with 60% (v/v) hydromethanol for 48 hours. Then it was filtered and evaporated to dryness under reduced pressure in a rotary vacuum evaporator at 40°C. The extract was stored at 4°C until being used.

### 2.3. GC-MS Analysis

The GC-MS analysis of the *Allium hirtifolium* extract was carried out by Gas Chromatography Mass Spectrometer Shimadzu QP 5050A. The GC conditions were splitless modes of injection, injector/interface temperature 250/260°C. Helium at a flow rate of 1.0 mL/min was employed as a carrier gas. The oven temperature was programmed as follows: 100°C for 3 minutes and gradually increased to 250°C for 15 minutes. The identification of the constituents was performed according to the Wiley and Nist mass spectral library. 

### 2.4. Bacterial Strains

The following strains were purchased from ATCC and tested in the screening.

*Gram-Positive Bacteria*: methicillin resistant Staphylococcus aureus (MRSA) ATCC 700698, methicillin sensitive Staphylococcus aureus (MSSA) ATCC 29247, Staphylococcus aureus ATCC 25923, Staphylococcus epidermidis ATCC 12228, Streptococcus pneumonia ATCC 10015. 

*Gram-Negative Bacteria*: *Escherichia coli* ATCC 25922, *Escherichia coli* O157:H7 ATCC 35159, *Salmonella typhimurium* ATCC 13311, *Proteus mirabilis* ATCC 29906, and* Klebsiella pneumoniae* ATCC 13883. 

All microorganisms were stored at −80°C as a stock and maintained at 4°C on Mueller-Hinton Agar (Merck, Germany).

### 2.5. Antibacterial Susceptibility Test

#### 2.5.1. Disk Diffusion Assay

The *in vitro* antimicrobial activity was screened by the disc diffusion method according to the protocol by Zaidan et al. [18] with minor modifications. Three hundred *μ*L of bacterial culture (suspended in tryptic soy broth) (Merck, Germany) adjusted to 0.5 McFarland standard was spread on Muller-Hinton agar plates evenly using a sterile swab and allowed to dry for 15 minutes. The different concentrations of extract (120, 60 mg/mL) filtered by 0.45 *μ*m millipore filters (Orange Scientific, Belgium) were impregnated on 6 mm sterile discs (Whatman paper number 1) with 20 *μ*L per disc. Then, the loaded discs were placed on the surface of inoculated medium.The plates were incubated at 37°C for 24 hours. At the end of incubation, the plates were examined and recorded for inhibition zone. Gentamycin (Sigma) was used as the positive control. The average of each zone of inhibition was calculated and recorded.

#### 2.5.2. Microdilution Broth Assay

The procedure was implemented as described by Khan et al. [19] with minor modifications. Different concentrations of extract in two-fold serial dilutions were prepared in tryptic soy broth. One hundred *μ*L of inoculum suspension with the optical density in the range of 0.08–0.10 (adjusted using spectrophotometer (Beckman Coulter Inc., Fullerton) at 600 nm or 0.5 Mcfarland standard (1.5 × 10^8^ CFU/mL)) was added into each well of a 96-well microtitre plate. Afterwards, 100 *μ*L of extract was added to the previous wells giving a final volume of 200 *μ*L. The 96-well microtiter plate was shaken for 20 seconds at 300 rpm and incubated at 37°C for 24 hours. The inoculum suspension was used as the negative control. Gentamycin (10 *μ*g/mL) was used as the positive control, while tryptic soy broth alone was used as blank. 

MIC is the lowest concentration which inhibits bacterial growth or it is the lowest concentration of the extract at which the microorganism does not demonstrate visible growth.

Confirmatory test, MBC, (minimum bactericidal concentration) was performed by loading 5 *μ*L of each well onto nutrient agar (Merck, Germany). MBC is the concentration at which there was no bacterial growth and the result was recorded.

### 2.6. Cells Viability Assay

#### 2.6.1. Cell Line

The Vero (African green monkey kidney cells) was purchased from ATCC and maintained in RPMI media (Sigma, USA) that incorporated with 1% Penicillin-Streptomycin, and Amphotericin B. The growth media was supplemented with 10% Fetal Bovine Serum (FBS) (Sigma, USA) and incubated inside 37°C, 5% CO_2_, and 95% of humidity.

#### 2.6.2. MTT Test

Monolayer cells were trypsinized, followed by washing with PBS (Sigma, USA), and seeded in each well of the 96-well flat-bottomed plates with density of 6.0 × 10^4^ cells/mL. After 24 hours of incubation time, the serial two-fold dilutions of extract were added to the confluent monolayer cells (except for the control) and the plate was incubated for further 24, 48, and 72 hours in a humidified incubator at 37°C and 5% CO_2_. The cells viability was determined by the MTT colorimetric technique [45] by adding 20 *μ*L of MTT (3-(4,5-dimethylthiazol-2-yl)-2,5-diphenyltetrazolium bromide) (Sigma, USA) solution (5 mg/mL in PBS) into each well. After 3 hours incubated in 37°C, 150 *μ*L of DMSO (dimethyl sulfoxide) (Merck, Germany) was added to each well in order to dissolve the MTT crystals. The plate was placed on the shaker for 15 minutes and the optical density was recorded at the wavelength of 590 nm (620 nm reference wavelength) using a microtitre plate reader (Tecan, Austria). The samples were performed in triplicate. The percentage of viable cells is calculated as (B/A) × 100, whereby A and B are the OD_590_ of untreated and treated cells, respectively [45]. The 50% cytotoxicity (CC_50_) of the test extract was determined from a curve of the cells viability (in percentage) versus the concentration of extract. CC_50_ is defined as the concentration that of reduces the OD_590_ of treated cells to 50% of that of untreated cells [46]. 

## 3. Results and Discussion

### 3.1. Chemical Composition of the Extract

The chemical constituents of the extract are presented in Table 1. In general, most chemical compositions found in the extract are fatty acids where the highest is 9-hexadecenoic acid, 18.09%, followed by 11, 14-eicosadienoic acid, 16.24%, and n-hexadecanoic acid for 15.26% while the other fatty acids are not more than 3%. Other organic compounds' percentages are less than 7%. The detection of fatty acids has also been reported by Ebrahimi et al. [17] who found that linolenic acid and linoleic acid with the percentages 14.66% and 78.88%, respectively are present in the *Allium hirtifolium* Boiss plant.

The individual chemical compounds were identified by matching their mass spectra of peaks with those obtained from the Wiley and Nist library mass spectra library. In a study performed by Ghodrati Azadi et al. [13], the presence of allicin (an organosulfur compound) compound was shown in *A. hirtifolium* Boiss by the TLC method. In contrast only a low percentage of sulfur compounds including s-methyl methanethiosulphonate, 2,4,5-trithiahexane, 2,4-dithiapentane, 2-pyridinethione and methane (chloromethylthio) (methylthio)- were detected in the current study. As reported by Iranshahi [47], these volatile sulfur compounds were formed due to the cleavage of odorless S-alk(en)yl cysteine sulfoxide flavor precursors by the enzymes alliinase and lachrymatory-factor synthase.

### 3.2. Antibacterial Effect of the Extract

The initial screening of antibacterial activity of the *Allium hirtifolium* Boiss hydromethanolic extract was conducted *in vitro* using the disk diffusion method. In general, the inhibition zones were observed for both gram-positive and gram-negative bacteria indicating their sensitivity to the extract as shown in Table 2. The inhibition zones ranged 11.5–21.5 mm and 10.0–16.5 mm for gram-positive and gram-negative bacteria, respectively. The extract also showed inhibitory activity on bacterial growth in a dose dependent manner. The hydromethanolic extract presented an antibacterial activity stronger than that of Gentamycin (positive control) against *Streptococcus pneumonia *ATCC 10015*, Escherichia coli *ATCC 25922, and *Escherichia coli* O157:H7 ATCC 35159. As shown in Table 3, the MIC and MBC values varied among the tested bacteria, and in most cases, the MIC values were lower than MBC values, indicating a bacteriostatic activity of the extract. 

In terms of the range of the inhibition zone as well as the MIC and MBC values, it was generally observed that the extract was more effective against gram-positive bacteria compared to the gram-negative bacteria which might be due to the differences in their cell wall structures. Specifically, gram-positive bacteria lack outer membrane while the outer membrane possessed by gram-negative bacteria might act as a barrier to many types of environmental substances which also include antibiotics [48]. The findings of the present study are in agreement with the previous report that showed that the gram-positive bacteria were more sensitive to the different types of *Allium roseum* extracts with the mean of growth inhibition zone ranged between 8 and 15 mm [49]. Chikwem et al. [50] also reported that the *Allium sativum* extract could inhibit the growth of gram-positive bacteria with a larger size of inhibition zone which was more than 20 mm in diameter by disk diffusion assay compared to gram-negative bacteria. This result was in contrast to other *Allium sativum* extracts, which mainly exhibited their antibacterial activity on gram-negative bacteria [51, 52]. Studies have shown that fatty acids, particularly methyl ester (FAME) found in plants such as *S. brachiate, *possess antibacterial and antifungal activities [53]. Therefore, we speculate that the antibacterial effect of *A. hirtifolium* Boiss extract might also be due to the presence of fatty acids such as 9-hexadecenoic acid,11,14-eicosadienoic acid, and n-hexadecanoic acid. 

### 3.3. Effect of the Extract on Normal Cells Viability

**Figure 1:**
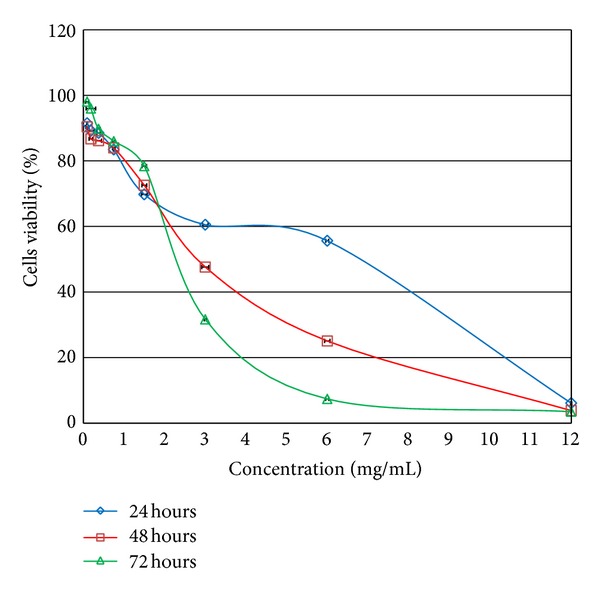
Effect of *Allium hirtifolium *Boiss. hydromethanolic extract on the viability of normal monkey kidney cell line (Vero) after 24, 48, and 72 hours of incubation time. Data show means ± SD of three replicates.

As shown in Figure 1, the hydromethanolic extract of Persian shallot was not toxic to the Vero cells at concentrations of 1.50 mg/mL and below (1 mg/mL) as more than 60% of cells remained viable at different incubation times 24, 48, and 72 hours (Figure 1). The extract also showed concentration and time dependent inhibition for the growth of the cells. As for the CC_50_, the values were 6.90, 2.90, and 2.60 mg/mL for 24, 48, and 72 hours, respectively, as indicated in Table 4.

With regard to antibacterial effect through microdilution broth assay, the minimum inhibitory concentration (MIC) of the extract against all gram-positive bacteria except for *Streptococcus pneumonia *was 1.88 mg/mL which is almost similar to nontoxic concentration to normal mammalian cells. As for other pathogens used in this study, the MIC and MBC values were higher than the nontoxic concentration to normal cells.However, this controversy might be solved if further study on the mode of actions of individual compounds of the extract is carried out [54] or some appropriate chemical and structure modifications are applied on the extract [55, 56]. Moreover, in a study by Levison [57], it was shown that the effective MIC and MBC values used *in vivo* were lesser than obtained values *in vitro*. Therefore, further investigation is required for the Persian shallot extract to be used as a safe antibacterial agent.

Azadi et al. [58] reported that the nontoxic concentration of chloroformic extract of *Allium hirtifolium* to normal mouse fibroblast cell line L929 was 1.00 mg/mL. Besides that, a previous study showed that the garlic and onion, which both belong to the genus *Allium hirtifolium*, exhibited a significant activity as cytoprotective agents on normal cells [59]. Cao et al. [60] also reported that DADS compound from *Allium sativum *has shown to be less harmful against normal cell line (type of normal cell used was not stated).

## 4. Conclusion

The present study revealed that the hydromethanolic extract of *Allium hirtifolium* Boiss exhibited antibacterial activity. In general, the results indicated that gram-positive bacteria appeared to be more sensitive to the extract compared with gram-negative bacteria. It was also shown to be noncytotoxic to normal cells at concentration below 1.50 mg/mL. More detailed studies such as separation of the hydromethanolic extract, isolation of single compound from the extract, and fractionation might be useful in the development of a Persian shallot-based medicine. Besides, further *in vivo* studies are also required to validate these *in vitro* observations.

## Figures and Tables

**Table 1:** Composition of the hydromethanolic extract of *Allium hirtifolium *Boiss bulbs.

Peak number	Compound	SI	Retention indices (type of column) (references)	MW	Percentage
1	2-pentanone,4-hydroxy-4-methyl	92	818 (methyl silicone) [20]	116	0.72
2	Acetic acid anhydride	95	699 (methyl silicone) [21]	88	4.06
3	2,3-Butanediol	97	793 (DB-5), 1492 (Carbowax 20 M) [22, 23]	90	3.16
4	1,3-Butanediol	98	784 (DB-Wax) [24]	90	2.39
5	2,4,5-Trithiahexane	85	1613 (Carbowax 20 M), 1098 (HP-1), 1106 (OV-1), 1211 (HP-5), 1666 (HP-INNOWax) [25–29]	140	2.03
6	Hexanoic acid	93	981 (HP-5MS) [30]	116	0.38
7	S-Methyl methanethiosulphonate	90	934 (HP-1) [29]	126	0.43
8	2,4-Dithiapentane	80	900 (HP-5MS), 871 (OV-1) [27, 31]	108	1.15
9	Benzene,1,2-Dimethoxy-4-(2-propenyl)-	90	1401 (DB-5) [32]	178	6.67
10	(Diisopropylamino)ethanol	74		145	0.38
11	Methyl P-vinylbenzoate	81		162	0.26
12	2-Pyridinethione	85		111	1.16
13	n-Decanoic acid	80	1347 (DB-5) [33]	172	0.26
14	Benzene,1,2-dimethoxy-4-(2-propenyl)-	80	1401 (DB-5) [32]	178	0.23
15	Pentadecanoic acid, 14-methyl-, methyl ester	96	1884 (DB-5) [34]	270	1.71
16	1,3-Dioxolane,2,4,5-trimethyl	84	745 (DB-5) [35]	116	5.22
17	2,4,5-Trithiahexane	85	1613 (Carbowax 20 M), 1098 (HP-1), 1106 (OV-1), 1211 (HP-5), 1666 (HP-INNOWax) [25–29]	140	1.16
18	Phenol-3,5-bis(1,1-dimethylethyl)-	93		206	1.42
19	s-Triazolo[4,3-a] Pyridine,7-methyl-	65		133	0.36
20	D-Glucitol, 1,4:3,6-dianhydro-	75		146	0.63
21	m-Dioxane, 2,4-dimethyl-	76	786 (OV-101) [36]	116	0.51
22	Methyl elaidate	95	2084 (DB-5) [33]	296	2.94
23	9-Octadecenoic acid, ethyl ester	90	2110 (DB-5) [37]	310	0.93
24	9,12-Octadecadienoic acid, methyl ester	94	2064 (HP-5MS) [38]	294	2.44
25	Hydroxymethylfurfurole	84	1812 (DB-Wax) [39]	126	1.90
26	9,12-Octadecadienoic acid, methyl ester	90	2064 (HP-5MS) [38]	294	1.17
27	Linolenic acid, methyl ester	85		292	0.38
28	Methyl 3-(3,5-di-tert-butyl-4-hydroxyphenyl) propionate	79		292	0.69
29	3,4,5-Trimethoxybenzoic acid, methyl ester	84		226	0.69
30	L-Glutamic acid	90		147	1.05
31	Methane,(chloromethylthio)-(methylthio)-	80		142	0.49
32	Tetradecanoic acid	84	2585 (DB-Wax) [39]	228	0.44
33	Phenol, nonyl	66	1720 (HP-5) [40]	220	0.12
34	Ethyl N-(O-anisyl)formimidate	67		179	0.34
35	Cinnamic acid	75	1386 (HP-1) [41]	148	0.26
36	n-Hexadecanoic acid	95	1962 (DB-5) [42]	256	15.26
37	1-Tetradecanol	80	1673(DB-5) [43]	214	0.35
38	Octadecanoic acid	87	2182 (DB-5) [33]	284	0.52
39	9-Hexadecenoic acid	93	1949 (HP-5) [44]	254	18.09
40	11,14-Eicosadienoic acid, methyl ester	92		322	16.24
41	Linolenic acid, methyl ester	91		292	1.38

**Table 2:** *In vitro* antibacterial activity of hydromethanolic extract of *Allium hirtifolium* Boiss against particular pathogens.

Pathogens	Diameter of inhibition zone (mm)		
	Concentration of extract (mg/mL)		Gentamycin^a^
	120^b^	60^c^	
Gram-positive bacteria			
MRSA ATCC 700698	18.17 ± 0.75	15.70 ± 0.52	23.50 ± 0.55
MSSA ATCC 29247	17.50 ± 0.55	15.30 ± 0.52	22.70 ± 0.52
*Staphylococcus aureus* ATCC 25923	15.00 ± 0.60	11.50 ± 0.55	23.70 ± 0.52
*Staphylococcus epidermidis *ATCC 12228	19.20 ± 0.75	15.80 ± 0.41	23.50 ± 0.55
*Streptococcus pneumonia *ATCC 10015	21.50 ± 0.84	18.30 ± 0.52	19.50 ± 0.84
Gram-negative bacteria			
*Escherichia coli *ATCC 25922	15.70 ± 0.52	14.00 ± 0.63	14.80 ± 0.41
*Escherichia coli* O157:H7 ATCC 35159	14.70 ± 0.52	11.50 ± 0.55	12.80 ± 0.41
*Salmonella typhimurium *ATCC 13311	14.70 ± 0.52	12.50 ± 0.84	22.50 ± 0.84
*Proteus mirabilis* ATCC 29906	11.30 ± 0.82	10.00 ± 0.63	14.50 ± 0.84
*Klebsiella pneumoniae* ATCC 13883	16.50 ± 0.84	14.20 ± 0.75	19.00 ± 0.63

Note: ^a^commercial antibiotic disk (Sigma) at 30 *μ*g/diskagainst gram-positive and gram-negative bacteria; ^b^potential of disk 2.4 mg/mL; ^c^potential of disk 1.2 mg/mL; *n* = 6; values are means ± SD of two replicates from three experiments.

**Table 3:** MIC and MBC (mg/mL) for antibacterial activity of *Allium hirtifolium* Boiss against particular pathogens.

Pathogens	Extract	
	MIC	MBC
Gram-positive bacteria		
MRSA ATCC 700698	1.88	7.50
MSSA ATCC 29247	1.88	7.50
*Staphylococcus aureus *ATCC 25923	1.88	3.75
*Staphylococcus epidermidis *ATCC 12228	1.88	15.00
*Streptococcus pneumonia *ATCC 10015	3.75	15.00
Gram-negative bacteria		
*Escherichia coli* ATCC 25922	7.50	30.00
*Escherichia coli* O157:H7 ATCC 35159	3.75	15.00
*Salmonella typhimurium *ATCC 13311	3.75	7.50
*Proteus mirabilis *ATCC 29906	7.50	30.00
*Klebsiella pneumonia *13883	3.75	15.00

Note: MIC: minimal inhibitory concentration; MBC: minimal bactericidal concentration.

**Table 4:** Minimum amounts of *Allium hirtifolium *hydromethanolic extract giving 50% of cell inhibition for normal monkey kidney cells (Vero).

Incubation time (hours)	IC_50_ value (mg/mL) (Vero cells)
24	6.90
48	2.90
72	2.60
